# The relationship between physical exercise and academic burnout in adolescents: the chain-mediated role of internet addiction and self-control

**DOI:** 10.3389/fpsyg.2025.1710564

**Published:** 2025-11-27

**Authors:** Jianbin Du, Jiahui Dong, Yan Shi, Enmeng Jiang, Lin Mo, Bingzhi Wan

**Affiliations:** 1 Ningbo Tech University, Ningbo, China; 2 School of Physical Education, Shaanxi Normal University, Xi'an, China; 3 Shaanxi Normal University Teacher Development College, Xi'an, China; 4 Faculty of Education, Shaanxi Normal University, Xi’an, China; 5 Department of Physical Education, Xidian University, Xi’an, China

**Keywords:** adolescents, physical exercise, academic burnout, internet addiction, self-control

## Abstract

**Background:**

Academic burnout represents a pressing issue among adolescents in China and has garnered increasing attention from scholars in the sport science domain. This study aims to investigate the underlying mechanisms linking physical exercise, internet addiction, self-control, and academic burnout, drawing on empirical survey data collected from adolescents in Shaanxi Province, China.

**Methods:**

This study employed a cross-sectional survey design, utilizing the *Physical Activity Rating Scale* (PARS-3), Chinese *Internet Addiction Test* (TAT), *Self-Control Scale* (SCS), and *Academic Burnout Inventory* (ABI) to collect data from 1,948 adolescents in Shaanxi Province (Mage = 13.74, SD = 1.37). Through descriptive analysis, we conducted statistical analyses on demographic variables. Utilizing Pearson correlation analysis, regression analysis, and chain mediation analysis, we constructed a relational model among variables.

**Results:**

The findings revealed significant pairwise correlations among Physical Exercise, Self-Control, Internet Addiction, and adolescent Academic Burnout. Specifically: Physical Exercise positively predicted Self-Control but negatively predicted Internet Addiction and Academic Burnout. Self-Control negatively predicted Internet Addiction and Academic Burnout. Internet Addiction positively predicted Academic Burnout.

**Conclusion:**

Self-Control and Internet Addiction independently mediated the relationship between Physical Exercise and adolescent Academic Burnout. Additionally, Self-Control and Internet Addiction jointly acted as chain mediators in this relationship.

## 1 Introduction

Against the backdrop of intensifying academic competition within China’s education system, adolescent academic burnout has become increasingly prevalent, posing a significant threat to teenagers’ holistic development (Chen and Chen, 2025). Academic burnout typically manifests through emotional exhaustion, diminished academic efficacy, and heightened disengagement from learning (Kiss and Pikó, 2025). It often forms a vicious cycle with internet addiction, each exacerbating the other, thereby further impairing adolescents’ psychosocial adjustment.

Guided by the positive youth development perspective and ecological systems theory, physical exercise has been identified as a multifaceted intervention that may mitigate academic burnout by enhancing self-control capacity and reducing internet addiction behaviors. Evidence from neurocognitive research indicates that regular physical activity can improve prefrontal cortex function, which in turn strengthens self-regulatory abilities. These improvements are reflected in better attentional focus, emotional stability, and time management skills, thereby alleviating emotional exhaustion and promoting academic performance. Moreover, participation in physical activities provides opportunities for in-person social interaction, achievement experiences, and bodily pleasure, serving as a functional alternative to online activities. This substitution effect helps reduce the risk of internet addiction and disrupts the self-perpetuating “stress - addiction - burnout” cycle.

Drawing on an integrated biopsychosocial perspective, this study aims to examine the underlying mechanisms through which physical exercise influences academic burnout, focusing specifically on the chain-mediated roles of self-control and internet addiction. By constructing and testing a sequential mediation model, this research seeks to elucidate the pathways through which exercise impacts burnout. The findings are expected to provide theoretical and practical insights for mitigating the negative consequences of academic pressure, promoting the integration of physical and academic education, and supporting the comprehensive development of adolescents.

### 1.1 Relationship between physical exercise and academic burnout in adolescents

Physical exercise is an active lifestyle behavior that plays a significant role in enhancing physical and mental health and improving behavioral habits. Yuan and Li (T.E.B.O.T.C.O.S.A.H, 2009) defined it as a scientific and rational form of physical activity that stimulates human organs, improves physiological functions, and thereby enhances overall health. Academic burnout refers to a psychological state in which students experience diminished enthusiasm for learning and reduced self-efficacy due to excessive academic pressure and workload. Schaufeli et al. (2002) categorized it into three dimensions: emotional exhaustion, learning detachment, and reduced sense of accomplishment. Although specific descriptions may vary, it is widely acknowledged that academic burnout primarily results from persistent stress and excessive demands (Wang, 2019). Academic burnout among adolescents has become a prominent psychological issue (Gao et al., 2025). Prolonged stress may lead to physical and mental exhaustion, alienation from teachers and peers, declining academic performance, and even trigger anxiety, sleep disorders, self-harm, or school dropout (Ling et al., 2014). Therefore, identifying effective methods to alleviate academic burnout is of great importance. Research indicates that physical exercise is an effective approach to mitigating academic burnout. The extent of its mitigating effect varies with the intensity of physical activity (Ling et al., 2014; Rahmati, 2015). Physical exercise can enhance physical fitness, improve emotional states, increase learning engagement (Zhong et al., 2025; Zhou, 2018), reduce stress sensitivity, and improve social adaptation and academic performance (Ji and Zheng, 2021; Mikkelsen et al., 2017) Overall, physical exercise has been shown to significantly negatively predict academic burnout, meaning that higher levels of physical exercise are associated with lower levels and a reduced probability of burnout. Therefore, the hypothesize 1 (H1) is proposed.

*H1*: Physical exercise is directly associated with academic burnout in adolescents.

### 1.2 The mediating role of internet addiction between physical exercise and academic burnout in adolescents

Adolescents represent a high-risk group for Internet addiction, which can extensively impair their physical, psychological, and social functioning. Specific consequences include vision and musculoskeletal problems, psychiatric comorbidities such as depression and anxiety, and in severe cases, even self-harm or suicidal behavior (Männikkö et al., 2015; Marchant et al., 2017). Internet addiction has become a global health issue, with prevalence rates ranging from 5.3 to 47.4%. Higher incidence among adolescents in Asia, particularly in China, is closely associated with the widespread availability of the Internet (Center, C.I.N.I, 2022; Tang et al., 2018). Studies suggest that physical exercise serves as an effective intervention for Internet addiction. Moderate-intensity aerobic exercise and activities such as Tai Chi have been shown to significantly reduce addictive behaviors (Fan et al., 2021; Yang and Zeng, 2017). The underlying mechanisms may involve enhanced neurotrophic factor expression, regulation of neurotransmitters, and improved central nervous system function (Akinci, 2021; Li J. B. et al., 2020). Simultaneously, Internet addiction has been found to be a significant positive predictor of academic burnout. Individuals with addiction tend to experience attentional deficits and reduced self-regulatory capacity, which contribute to diminished learning motivation and impaired self-efficacy (Akinci, 2021; Zhang, 2022). According to cognitive processing models, the attentional bias developed by addicts can interfere with normal learning processes and increase the risk of burnout (Greenfield, 2023; Scimeca et al., 2017). Therefore, physical exercise may alleviate academic burnout by reducing Internet addiction. Therefore, the H2 is proposed.

*H2*: Internet addiction plays a mediating role between physical exercise and academic burnout.

### 1.3 The mediating role of self-control between physical exercise and academic burnout in adolescents

Self-control refers to an individual’s ability to consciously regulate impulses, habits, or automatic responses in order to align behavior with social norms and long-term goals (de Ridder et al., 2012) As a psychologically malleable trait with lifelong plasticity, self-control exerts positive influences on cognition, emotion, and behavior, and has attracted widespread research interest in recent years. Based on the Strength Model of Self-control, Temporal Motivation Theory, and conceptual models of academic burnout, this study proposes that self-control may serve a mediating role between physical exercise and academic burnout (Wei, 2023) First, physical exercise is significantly associated with self-control capacity. According to the Strength Model of Self-control, although self-control temporarily depletes mental resources, sustained exercise can help recover and strengthen such resources—much like muscle training—ultimately enhancing self-control abilities (Finley and Schmeichel, 2019) Empirical studies have also indicated that physical exercise can significantly and positively predict self-control (Cao and Gao, 2023). Second, academic burnout is regarded as a negative psychological state resulting from prolonged academic pressure (Qian et al., 2015). Strong self-control facilitates the development of patience and perseverance, enabling individuals to respond more proactively to academic demands, thereby reducing burnout-related behaviors (Qin et al., 2020). Research has demonstrated a significant negative correlation between self-control and academic burnout: students with higher self-control exhibit lower levels of academic burnout, whereas those with insufficient self-control are more prone to burnout (Chen et al., 2022; Kühnel et al., 2018). Thus, academic burnout can be viewed as a manifestation of self-control failure (Wu et al., 2024), and self-control is considered a negative predictor of academic burnout. In summary, self-control may play a mediating role in the relationship between physical exercise and academic burnout. Therefore, the H3 is proposed.

*H3*: Self-control mediates the relationship between physical exercise and academic burnout in adolescents.

### 1.4 The serial mediating roles of self-control and internet addiction between physical exercise and academic burnout in adolescents

Current research indicates that self-control and Internet addiction each serve as independent mediators between physical exercise and academic burnout, with close interrelationships suggesting they may form a multiple mediation pathway. According to the protective-risk factor framework, self-control—as a protective factor—can effectively mitigate the negative impacts of risk factors such as Internet addiction (Baumeister and Tice, 2007; Kim et al., 2017). Strong self-control ability helps individuals regulate emotions and behaviors, thereby reducing excessive Internet use. Grounding in Self-Determination Theory, physical exercise can enhance intrinsic motivation and self-worth by satisfying basic psychological needs such as autonomy, competence, and relatedness. Self-control plays a crucial role in this process by helping individuals maintain exercise behavior and achieve a sense of accomplishment, which further strengthens self-regulatory capacity. However, Internet addiction may deplete self-control resources, resulting in self-regulatory failure and a subsequent vicious cycle of addiction (Baumeister and Tice, 2007). Studies have shown that physical exercise, such as running, can effectively improve self-control (Xie, 2013; Zhang et al., 2018), which may in turn indirectly suppress addictive online behaviors. Although existing studies have established relationships between physical exercise and Internet addiction, Internet addiction and academic burnout, and self-control and academic burnout, the underlying mechanisms among these four variables remain underexplored. Therefore, this study treats physical exercise as the independent variable and academic burnout as the dependent variable, with Internet addiction and self-control included as serial mediators, aiming to elucidate the complex pathways through which these variables interact. Based on the above theoretical and empirical evidence, Hypothesis H4 is proposed.

*H4*: Self-control and Internet addiction play serial mediating roles between physical exercise and academic burnout in adolescents.

**Figure 1:**
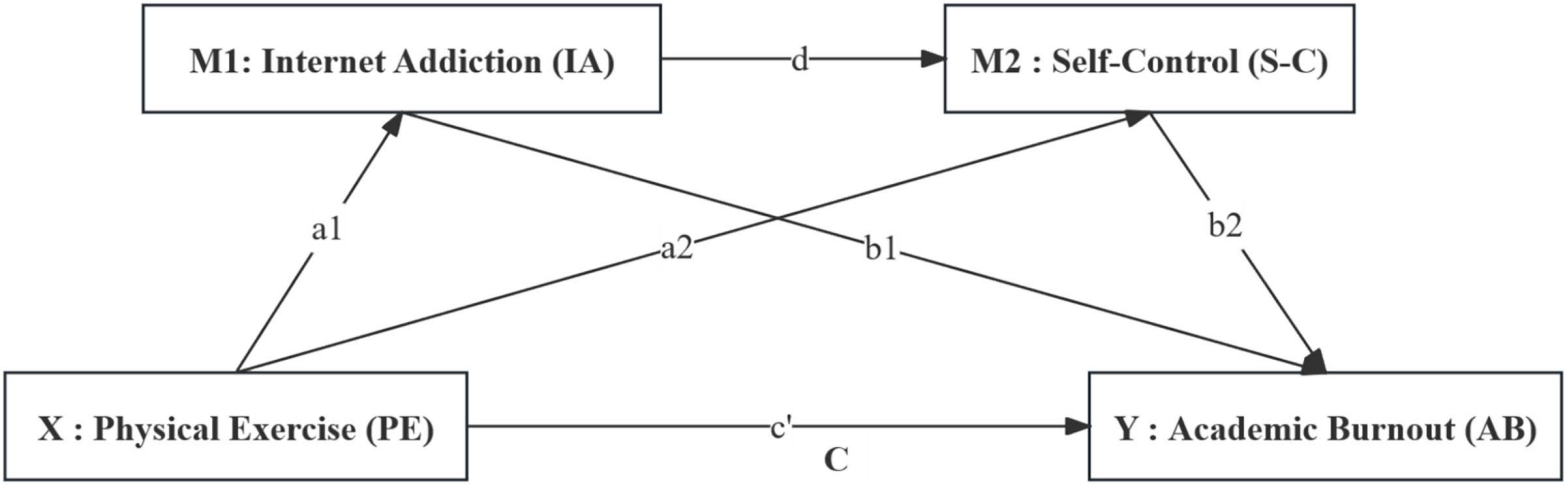
Hypothesized model diagram of physical exercise affecting academic burnout.

In summary, this study introduced Internet Addiction and Self-Control factors between Physical Exercise and Academic Burnout and constructed a chain mediation model as shown in Figure 1.

## 2 Methods

### 2.1 Participants

This research employs a cluster random sampling method, selecting 16 middle schools from Shaanxi Province, comprising 9 urban schools and 7 rural schools, as the subjects of investigation. The sampling strategy accounts for both geographical distribution and school types, thereby ensuring that the sample largely reflects the demographic characteristics of the student population in the region. It is important to note that, due to the cluster format of data collection, there may be within-group correlation in student responses within the same school. Given the limitations of the research methods and data structure, this study did not utilize a hierarchical linear model to statistically adjust for the clustering effect; however, this approach may be considered and analyzed in future research.

Data collection was conducted via both online (using Wenjuanxing, a widely-used survey platform in China) and offline questionnaires. Ethical clearance was granted by the Ethics Committee of Shaanxi Normal University (Approval No. 202418026). To ensure ethical compliance, the research team—consisting of the principal investigator and trained teachers—obtained approval from school administrators and collaborating instructors. Participants were surveyed during 15-min class breaks in quiet classroom settings. Prior to administration, students were informed of the research purpose, voluntary participation, anonymity, the importance of truthful responses and participants could withdraw at any time. Online surveys were completed via smartphone QR code scanning.

Following a 14-day data collection period, 2,212 questionnaires were returned. The survey sample capacity is measured beforehand, using the initial sample capacity calculation formula:


$$n=\frac{Z^{2}p(1-p)}{e^{2}}$$

Where Z = 1.96 (95% confidence level), *p* = 0.5 (maximum variance), and e = 3% (precision, the margin of error). This study will take the 95% confidence interval, the permissible margin of error is not more than 3%, substituting into the formula:

$n=\frac{1.960^{2}*0.5(1-0.5)}{0.03^{2}}$ *=* 1067.11.

The initial sample size is determined to be 1,068. According to the total sample size of the population in the research area, the formula is adopted:


$$n_{\text{adjust}}=\frac{\mathrm{nN}}{n+N-1}$$

Where N = 2.074 million people (overall sample size), according to Shaanxi Education Overview released by Shaanxi Provincial Education Department on May 24, 2024 (Government, E.D.O.P, 2024), the total number of middle and high school students is 2.074 million, and the data is brought into the formula:

$n_{\text{adjust}}=\frac{1067.11*2074000}{1067.11+2074000-1}$ ≈ 1,068.

The minimum valid sample size for this study was determined as1,068, ensuring sufficient statistical power. Among the 2,212 returned questionnaires, 264 invalid responses were excluded based on predefined criteria: (1) Completion time < 240 s, (2) Incomplete demographic information, (3) Missing values (>10% unanswered items), (4) Outliers (responses exceeding ±2 *SD*s from the mean).

This yielded a final valid sample of 1,948 participants (*Mage* = 13.74, *SD* = 1.37; see Table 1 for details), achieving an 88.07% valid response rate.

**Table 1:** List of basic information of valid subjects (*n* = 1,948).

Variable	Category	*N*	Percentage (%)
Gender	Boys	959	49.2%
	Girls	989	50.8%
Grade	Junior high school	1,199	61.6%
	Senior high school	749	38.4%
Body mass index	BMI<18.5	705	36.2%
	18.5 ≤ BMI<24	930	47.7%
	24 ≤ BMI	313	16.1%
Residential area	Urban	1,128	57.9%
	Rural	820	42.1%

BMI: Body mass index.

### 2.2 Measures

#### 2.2.1 Physical exercise scale (PARS-3)

The revised PARS-3 by Liang (1994) was administered to assess participants’ exercise levels. This scale evaluates exercise volume through three dimensions: intensity, frequency, and duration, using a 5-point Likert scale. Total exercise volume was calculated using the formula: Exercise Volume = Frequency × (Duration − 1) × Intensity. In this study, the *Kolmogorov–Smirnov (K–S)* test indicated a non-normal distribution (*p* < 0.001, *df* = 1,948), and the overall Cronbach’s *α* coefficient was 0.85, demonstrating good reliability.

#### 2.2.2 Internet addiction test (TAT)

The revised TAT was employed to measure Internet Addiction among adolescents (Liang, 1994; Young, 1998). The 20-item instrument adopts a 5-point Likert scale (1 = Strongly disagree, 5 = Strongly agree), with higher total scores reflecting greater Internet Addiction severity. The scale demonstrated excellent internal consistency in this study (*Cronbach’s α* = 0.914).

#### 2.2.3 Self-control scale (SCS)

A domestically adapted SCS (Tan and Guo, 2008) was utilized, comprising a total of 19 items across five dimensions: impulse inhibition (6 items), health habits (3 items), resistance to temptation (4 items), concentration on work (3 items), and abstinence from entertainment (3 items). This scale demonstrates strong reliability and validity in domestic contexts. It employs a 5-point Likert scoring system, where 1 represents “not at all” and 5 signifies “completely.” Notably, items 2, 3, 4, 6, 7, 8, 9, 10, 12, 13, 15, 16, 17, 18, and 19 are designated as reverse-scoring questions, and appropriate conversions were applied during the scoring process. Once all items were scored in the same direction, a higher total score indicated a greater level of self-control in the individual. The *Kolmogorov–Smirnov (K–S)* test revealed non-normality (*p* < 0.001, *df* = 1,948), and the scale achieved acceptable reliability (*Cronbach’s α* = 0.781). Previous validation studies in domestic populations have confirmed its robust psychometric properties.

#### 2.2.4 Academic burnout inventory (ABI)

The ABI developed by Hu and Dai (2007) was adopted to assess Academic Burnout across four dimensions: low efficacy (5 items), emotional exhaustion (6 items), physical depletion (5 items), and student-teacher alienation (5 items). The 21-item questionnaire employs a 5-point Likert scale (1 = Strongly disagree, 5 = Strongly agree). Nonparametric testing showed significant deviation from normality (*p* < 0.001, *df* = 1,948), with satisfactory internal consistency (*Cronbach’s α* = 0.817). Confirmatory factor analysis (*CFA*) demonstrated adequate model fit: *χ^2^/df* = 1.318, *RMSEA* = 0.06, *NFI* = 0.949, *CFI* = 0.955, *IFI* = 0.955, *PNFI* = 0.810, confirming strong construct validity.

## 3 Results

### 3.1 Common method bias test

Since all variables in this study were measured using self-reported data, common method bias might have been a concern. To address this, Harman’s single-factor test was conducted. An exploratory factor analysis without rotation was performed on all variables. The results showed that there were 12 factors with eigenvalues greater than 1, and the first factor accounted for 26.60% of the variance, which is significantly below the critical threshold of 40%. This indicates that common method bias did not significantly affect the results of this study.

### 3.2 Descriptive statistics and correlations analyses

This study investigated the current status of adolescents’ participation in sports and their purposes for using the internet (see Table 2). Regarding sports participation, interest in badminton was significantly higher among different gender and grade groups compared to other sports, while tennis had the lowest participation rate, accounting for no more than 5% of respondents. As for internet usage, socializing and watching videos were the primary purposes, with both exceeding 60%. Notably, over 65% of adolescents reported watching videos as their main online activity.

**Table 2:** List of sports program preferences and purpose of accessing the internet by gender and grade group (*n* = 1,948).

SE	Boys (*n* = 959)		Girls (*n* = 989)		Junior (*n* = 1,190)		Senior (*n* = 758)	
	Dislike	Like	Dislike	Like	Dislike	Like	Dislike	Like
Soccer ball	792 (82.6%)	167 (17.4%)	851 (86%)	138 (14%)	997 (83.8%)	193 (16.2%)	646 (85.2%)	112 (14.8%)
Soccer	810 (84.5%)	149 (15.5%)	822 (83.1%)	167 (16.9%)	1,006 (84.5%)	184 (15.5%)	626 (82.6%)	132 (17.4%)
Basketball	715 (74.5%)	244 (25.5%)	752 (76.0%)	237 (24.0%)	905 (76.1%)	285 (23.9%)	562 (74.1%)	196 (25.9%)
Ping-pong	675 (70.4%)	284 (29.6%)	723 (73.1%)	266 (26.9%)	848 (71.3%)	342 (27.2%)	550 (72.6%)	208 (27.4%)
Squash	913 (95.2%)	46 (4.8%)	948 (95.9%)	41 (4.1%)	1,131 (95.0%)	59 (5.0%)	730 (96.3%)	28 (3.7%)
Shuttlecock	366 (38.2%)	593 (61.8%)	374 (37.8%)	615 (62.2%)	456 (38.3%)	734 (61.7%)	284 (37.5%)	474 (62.5%)
Run	619 (64.5%)	340 (35.5%)	653 (66.0%)	336 (34.0%)	785 (66.0%)	405 (34.0%)	487 (64.2%)	271 (35.8%)
Group of dancers	848 (88.4%)	111 (21.6%)	812 (82.1%)	177 (17.9%)	1,022 (85.9%)	168 (14.1%)	638 (84.2%)	120 (15.8%)

IP	NP	P	NP	P	NP	P	NP	P
Socialize	355 (37.0%)	604 (63.0%)	362 (36.6%)	627 (63.4%)	448 (37.6%)	742 (62.4%)	269 (35.5%)	489 (64.5%)
Browse the web	574 (59.8%)	385 (40.2%)	579 (58.5%)	392 (39.6%)	716 (60.2%)	474 (39.8%)	455 (60.0%)	303 (40.0%)
Play a game	538 (56.1%)	421 (43.9%)	584 (59.0%)	405 (41.0%)	656 (55.1%)	534 (44.9%)	466 (61.5%)	292 (38.5%)
Watch the video	299 (31.2%)	660 (68.8%)	300 (30.3%)	689 (69.7%)	368 (30.9%)	822 (69.1%)	231 (30.5%)	527 (69.5%)
Take an online course	593 (61.8%)	366 (38.2%)	603 (61.0%)	386 (39.0%)	730 (61.3%)	460 (38.7%)	466 (61.5%)	292 (38.5%)

SE, sports event; IP, internet purpose; NP, non-participation; P, participation.

The correlation analysis (see Table 3) revealed the following relationships: Physical Exercise was significantly negatively correlated with Academic Burnout (*r* = −0.119, *p* < 0.01) and Internet Addiction (*r* = −0.124, *p* < 0.01), but significantly positively correlated with Self-Control (*r* = 0.116, *p* < 0.01). Academic Burnout was significantly positively correlated with Internet Addiction (*r* = 0.449, *p* < 0.01) and significantly negatively correlated with Self-Control (*r* = −0.484, *p* < 0.01). Internet Addiction was significantly negatively correlated with Self-Control (*r* = −0.494, *p* < 0.01). These results suggest that an increase in Physical Exercise or enhanced Self-Control can mitigate Academic Burnout, thereby reducing its severity. Conversely, an increase in Internet Addiction is associated with a corresponding rise in Academic Burnout (Sensitivity analyses, which included Spearman correlation analysis and robust standard error estimation, were conducted to assess the robustness of the study results. These findings aligned with the conclusions drawn from the primary analysis, suggesting that the results were not influenced by particular distribution assumptions. The outcomes of these supplementary analyses are detailed in the appendices and Supplementary material).

**Table 3:** Correlation analysis among all variables.

Variable	M ± SD	1	2	3	4
Physical exercise (1)	3.037 ± 0.779	1	–	–	–
Internet addiction (2)	1.894 ± 0.620	−0.124**	1	–	–
Self-control (3)	3.317 ± 0.353	0.116**	−0.484**	1	–
Academic burnout (4)	2.296 ± 0.497	−0.119**	0.449**	−0.494**	1

The mean (M) and standard deviation (SD), *p < 0.05; **p < 0.01; same below.

### 3.3 Testing and analysis of the serial mediation model

To investigate the intrinsic relationships among Physical Exercise, Internet Addiction, and Self-Control in predicting Academic Burnout, regression analyses were conducted using these three factors as independent variables and Academic Burnout as the dependent variable, based on preliminary correlation analyses. The statistical results are presented in Table 4.

**Table 4:** Regression analysis results.

Regression equation		Overall fit index				Significance of regression coefficient		
Independent variable (IV)	Dependent variable (DV)	*R*	*△R2*	*F*	*p*	*β*	*t*	*p*
PE	AB(1)	0.119	0.014	28.082	<0.01	−0.076	−5.299	<0.001
PE	AB(2)	0.454	0.205	252.273	<0.01	−0.041	−3.168	<0.01
IA						0.354	21.673	<0.001
PE	AB(3)	0.498	0.247	320.742	<0.01	−0.04	−3.172	<0.01
S-C						−0.685	−24.590	<0.001
PE	AB(4)	0.551	0.303	282.761	<0.01	−0.028	−2.310	<0.05
IA						0.217	12.481	<0.001
S-C						−0.504	−16.527	<0.001
PE	IA	0.124	0.015	30.426	<0.01	−0.099	−5.516	<0.001
	S-C	0.116	0.013	26.552	<0.01	0.053	5.153	<0.001
IA	AB	0.449	0.201	492.221	<0.01	0.360	22.186	<0.001
	S-C	0.484	0.233	594.073	<0.01	−0.849	−24.374	<0.001
S-C	AB	0.494	0.244	628.497	<0.01	−0.695	−25.070	<0.001

PE, physical exercise; IA, internet addiction; S-C, self-control; AB, academic burnout.

#### 3.3.1 Regression model fit results

Hierarchical regression analysis was conducted to examine the predictive impact of Physical Exercise, Internet Addiction, and Self-Control on Academic Burnout. Initially, Model 1 solely incorporated Physical Exercise as an independent variable, revealing a variance explanation of *ΔR^2^* = 0.014 (*F* = 28.082, *p* < 0.01). Upon the addition of the Internet Addiction variable in Model 2, the variance explanation increased significantly to *ΔR^2^* = 0.205 (*F* = 252.273, *p* < 0.01), with Internet Addiction accounting for 19.1% of the variance. Subsequently, Model 3 integrated Self-Control variables, resulting in a variance explanation of *ΔR^2^* = 0.247 (*F* = 320.742, *p* < 0.01), where Self-Control contributed to 23.3% of the variance. The final Model 4 encompassed all predictors, culminating in an overall variance explanation of *ΔR^2^* = 0.303 (*F* = 282.761, *p* < 0.01).

#### 3.3.2 Direct effects between variables

Physical Exercise exhibits a significant negative predictive effect on Academic Burnout (*β* = −0.076, *t* = −5.299, *p* < 0.001). Specifically, a 1 standard deviation increase in physical exercise corresponds to a 0.076 standard deviation decrease in academic burnout.

Physical Exercise is significantly negatively correlated with Internet Addiction (*β* = −0.099, *t* = −5.516, *p* < 0.001), indicating that a 1 standard deviation increase in Physical Exercise results in a 0.099 standard deviation decrease in Internet Addiction.

Physical Exercise is significantly positively correlated with self-control (*β* = 0.053, *t* = 5.153, *p* < 0.001), with Self-Control increasing by 0.053 standard deviations for each standard deviation increase in Physical Exercise.

Internet Addiction has a significant positive predictive effect on Academic Burnout (*β* = 0.360, *t* = 22.186, *p* < 0.001), whereby Academic Burnout increases by 0.360 standard deviations for each standard deviation of Internet Addiction.

Internet Addiction significantly negatively predicts Self-Control (*β* = −0.849, *t* = −24.374, *p* < 0.001), leading to a 0.849 standard deviation decrease in Self-Control for each standard deviation increase in Internet Addiction.

Self-Control significantly negatively predicts Academic Burnout (*β* = −0.695, *t* = −25.070, *p* < 0.001), resulting in a 0.695 standard deviation decrease in Academic Burnout for each standard deviation increase in Self-Control.

#### 3.3.3 Effect of change in multivariate model

In the complete model 4, after controlling for other variables, each 1 standard deviation increase in physical activity is associated with a direct reduction in academic burnout of 0.028 standard deviations (*β* = −0.028, *t* = −2.31, *p* < 0.05). Conversely, academic burnout increases by 0.217 standard deviations for each standard deviation of Internet addiction (*β* = 0.217, *t* = 12.48, *p* < 0.001). Additionally, academic burnout decreases by 0.504 standard deviations for each standard deviation of self-control (*β* = −0.504, *t* = −16.527, *p* < 0.001).

All statistical analyses demonstrated significance (*p* < 0.05).

Following the mediation analysis procedures proposed by Zhonglin Wen et al.:

Step 1: The regression coefficient (c) of Physical Exercise on Academic Burnout was examined (*c* = −0.076, *t* = −5.299, *p* < 0.001). The results confirmed the existence of a significant mediation effect between Physical Exercise and Academic Burnout.

**Figure 2:**
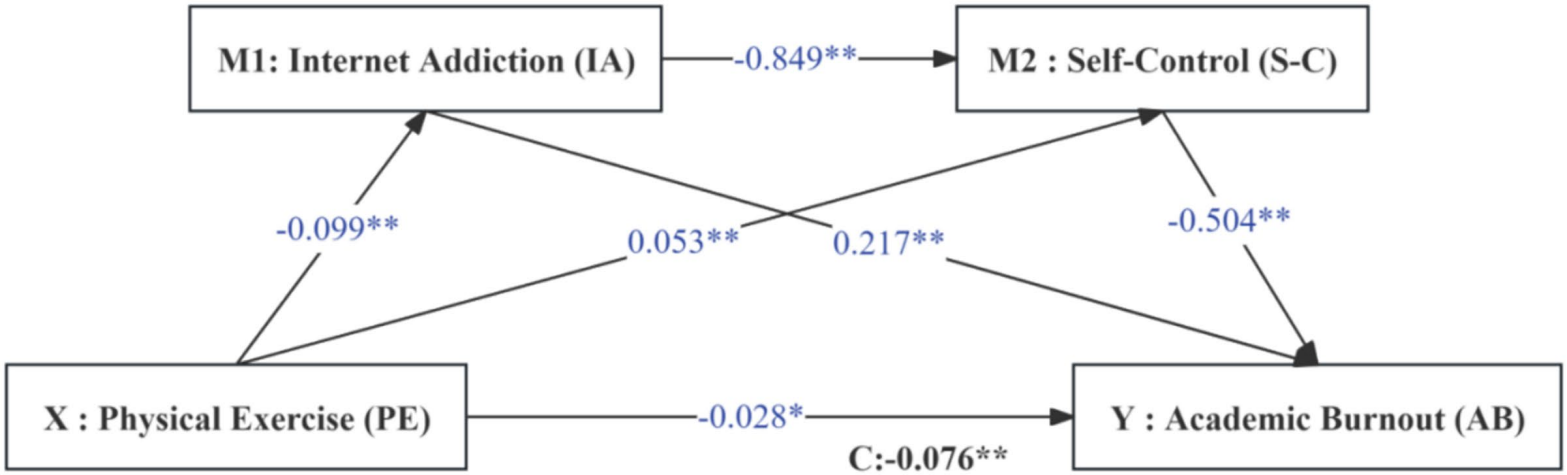
The influence of physical exercise on academic burnout.

Step 2: Sequential testing revealed significant coefficients for all paths: a_1_ = −0.099 and a_2_ = 0.053 (Physical Exercise to mediators); b_1_ = 0.217 and b_2_ = −0.504 (mediators to Academic Burnout). All four coefficients were statistically significant, indicating significant indirect effects. The resulting model and the path relationships among the variables are shown in Figure 2.

Step 3: To examine the chain mediation effects of Internet Addiction and Self-Control, the Bootstrap method was employed for chain mediation effect testing. Model 6 was selected, with a sample size of 5,000, and the significance of the mediation effects was assessed at a 95% confidence interval (CI). The results (see Table 5) showed that:

1. The mediation effect of Internet Addiction was −0.214, with a 95% CI [−0.030, −0.013].
2. The mediation effect of Self-Control was −0.013, with a 95% CI [−0.022, −0.004].
3. The chain mediation effect of both variables was −0.014, with a 95% CI [−0.019, −0.008].

**Table 5:** Bootstrap analysis and its effect size for significance test of intermediary effect (*n* = 1,948).

Impact pathways	Effect	Boot standard error	Boot95% CI lower limit	Boot95% CI cap	Relative mediation effect
Ind1	−0.021	0.004	−0.030	−0.013	28.12%
Ind2	−0.013	0.005	−0.022	−0.004	17.08%
Ind3	−0.014	0.003	−0.019	−0.008	17.74%
Total indirect effect	−0.048	0.008	−0.064	−0.032	62.94%
Direct effect	−0.028	0.012	−0.052	−0.004	37.06%
Aggregate effect	−0.076	0.014	−0.104	−0.048	
C1 (Ind1 - Ind2)	−0.008	0.007	−0.022	0.004	
C2 (Ind1 - Ind3)	−0.008	0.003	−0.014	−0.002	
C3 (Ind2 - Ind3)	0.0005	0.006	−0.001	0.012	

PE, physical exercise; IA, internet addiction; S-C, self-control; AB, academic burnout. Ind1 = PE - IA - AB; Ind2 = PE - SC - AB; Ind3 = PE - IA - SC - AB. C1 = Ind1 - Ind2; C2 = Ind1 - Ind3; C3 = Ind2 - Ind3.

All confidence intervals excluded zero, confirming significant mediation effects.

Further Decomposition of Effect Sizes: The direct effect of Physical Exercise on Academic Burnout was −0.028, while the total indirect effect, derived from the sum of the mediation effects across the three pathways, was −0.048. The total effect, calculated as the sum of the direct and total indirect effects, amounted to −0.076. The relative contribution of each mediation pathway to the total effect was determined by dividing the individual mediation effects by the total effect. The proportions for the three mediation pathways were 28.12, 17.08, and 17.74%, respectively.

The findings indicate that a one standard deviation increase in Physical Exercise was associated with a reduction in Academic Burnout by −0.076 standard deviations. Of this reduction, −0.048 standard deviations were attributable to indirect mediation effects. Specifically: The mediation effect of Internet Addiction exerted a negative influence, accounting for 28.12% of the total indirect effect. Similarly, the mediation effect of Self-Control also demonstrated a negative influence, contributing 17.08% to the total effect. The interaction between Internet Addiction and Self-Control further contributed a negative inhibitory effect, representing 17.74% of the total effect.

Comparison of Mediation Effects: A comparative analysis of the three mediation pathways revealed that, in terms of individual mediation effects, the negative influence of Self-Control was significantly stronger than that of Internet Addiction (*E* = −0.008, *LLCI* = −0.022, *ULCI* = 0.004). The chain mediation effect involving both variables was weaker than their respective individual mediation effects. Specifically: The chain mediation effect significantly differed from the mediation effect of Internet Addiction (*E* = −0.008, *LLCI* = −0.014, *ULCI* = −0.002). It also significantly differed from the mediation effect of Self-Control (*E* = 0.0005, *LLCI* = −0.0010, *ULCI* = 0.012).

## 4 Discussion

This study elucidates the chain mediation effects of Internet Addiction and Self-Control in the relationship between Physical Exercise and Academic Burnout among adolescents, specifically junior high and high school students. It represents an exploratory effort to understand how Physical Exercise can serve as a preventive measure against Academic Burnout. The research contributes to theoretical knowledge by enhancing the understanding of the mechanisms underlying Academic Burnout and by deepening insights into the relationship between Physical Exercise and Academic Burnout in this demographic. Furthermore, it sheds light on how Physical Exercise can improve psychological well-being. It offers valuable insights for promoting Physical Exercise, decreasing Internet Addiction, improving Self-Control, and averting Academic Burnout, thus presenting noteworthy practical implications.

### 4.1 Relationship between physical exercise and academic burnout

This study examined the intrinsic relationship between Physical Exercise and Academic Burnout among adolescents. The findings indicate that Physical Exercise significantly predicts a decrease in adolescent Academic Burnout (*β* = −0.076, *p* < 0.001). Although the direct effect size is relatively modest (*β* = −0.076 in model 1 and *β* = −0.028 in model 4), it exerts a substantial influence through two indirect pathways. First, Physical Exercise mitigates Internet Addiction (*β* = −0.099), and second, it enhances Self-Control (*β* = 0.053). This model suggests that the beneficial impact of Physical Exercise on Academic Burnout primarily operates through the enhancement of intermediate psychological mechanisms rather than through direct effects. This finding validates Hypothesis H1 and aligns with previous research (Cheung and Li, 2019; Wang et al., 2023). According to the Theory of Planned Behavior proposed by Ajzen (1991), individual behavioral intentions are influenced by three core elements: attitude, subjective norms, and perceived behavioral control. First, the more positive an individual’s attitude toward a specific behavior, the stronger their behavioral intention. Second, the more positive the perceived subjective norms, the stronger the behavioral intention. Finally, when both attitude and subjective norms are positive, an individual’s sense of behavioral control is significantly enhanced, thereby strengthening their behavioral intention. This theoretical framework provides important support for understanding the relationship between Physical Exercise and Academic Burnout.

From a psychological perspective, Physical Exercise, as an active health behavior intervention, not only cultivates adolescents’ perseverance and stress resistance but also significantly enhances their self-efficacy and behavioral activation. When adolescents develop regular Physical Exercise habits, their time management and self-regulation abilities are significantly improved. These improvements can extend to the academic domain through the skill transfer effect (James et al., 2023). Specifically, psychological qualities such as goal setting, persistence, and frustration tolerance, fostered through Physical Exercise, can effectively reduce the incidence of Academic Burnout. Therefore, this study supports the view that Physical Exercise is an effective intervention for Academic Burnout, which is consistent with the mainstream conclusions in the current academic literature (Takehara et al., 2019).

### 4.2 The mediating role of internet addiction

This study, based on the dual-pathway model, elucidates the mechanisms through which Physical Exercise influences adolescent Academic Burnout. The results demonstrate that the negative predictive effect of Physical Exercise on Academic Burnout can be achieved through both direct and indirect pathways, specifically the “Physical Exercise → Internet Addiction → Academic Burnout” pathway (*β* = − 0.041, *p* < 0.01; *β* = 0.354, *p* < 0.001; mediation effect size = −0.021). This finding confirms Hypothesis H2. From a neurobiological perspective, Physical Exercise can improve the structure and function of brain regions such as the prefrontal cortex and striatum, bidirectionally regulating the expression levels of dopamine and its receptors, thereby effectively reducing the incidence of Internet Addiction (Li S. et al., 2020). The results of this study support this view, showing a significant negative correlation between adolescent Physical Exercise and Internet Addiction (*r* = −0.124, *p* < 0.01). Moreover, different types of Physical Exercise were found to significantly reduce the severity of Internet Addiction.

From a behavioral psychology perspective, Internet Addiction is a key risk factor for adolescent Academic Burnout. According to Self-regulation Theory, adolescents with higher levels of Internet Addiction often exhibit poorer Self-Control and time management skills. These deficits in self-regulation significantly increase the risk of Academic Burnout (Tomaszek and Muchacka-Cymerman, 2022). The study’s findings revealed a substantial positive association between Internet Addiction and Academic Burnout (*r* = 0.449, *p* < 0.01) with a moderate effect size (*β* = 0.360). This relationship retained statistical significance in model 4 (*β* = 0.217). Adolescents with severe Internet Addiction symptoms not only reduce their time investment in learning but also engage in task avoidance behaviors, thereby exacerbating the severity of Academic Burnout.

Notably, as an active behavioral intervention, Physical Exercise can reduce adolescents’ internet usage time through the displacement effect while simultaneously fostering their self-management skills and goal-directed behaviors. Studies have demonstrated that regular engagement in Physical Exercise is associated with lower levels of Internet Addiction, which in turn correlates with reduced Academic Burnout, suggesting a potential preventive effect (Chen et al., 2022). This finding offers significant theoretical and practical evidence for utilizing Physical Exercise as a means to intervene in adolescent Internet Addiction and Academic Burnout.

### 4.3 The mediating role of self-control

The results of this study indicate that the negative predictive effect of Physical Exercise on Academic Burnout can be achieved through both direct (*β* = −0.076, *p* < 0.001) and indirect pathways, specifically the “Physical Exercise → Self-Control → Academic Burnout” pathway (*β* = − 0.04, *p* < 0.01; *β* = − 0.685, *p* < 0.001 mediation effect size = −0.013). This finding supports Hypothesis H3 and aligns with previous research (Diamond and Ling, 2016). From the perspective of neuroplasticity, regular Physical Exercise significantly enhances the executive functions of the prefrontal cortex, particularly core components such as inhibitory control and cognitive flexibility, thereby improving an individual’s Self-Control capacity (Tangney et al., 2004). This finding is consistent with the experimental results of Mark (Muraven, 2010), who demonstrated that aerobic exercise and endurance training significantly improve Self-Control capacity compared to sedentary behavior (*p* < 0.01). The regression coefficient analysis in this study (*r* = 0.116, *p* < 0.01) further validates this perspective, providing empirical support for the role of Physical Exercise in enhancing Self-Control capacity.

According to the self-regulatory failure theory of academic procrastination, self-control is a crucial protective factor against Academic Burnout. Adolescents who engage in regular Physical Exercise can enhance their Self-Control by developing exercise plans, monitoring exercise intensity, and adjusting their exercise behaviors. This enhancement in Self-Control can transfer to academic contexts, leading to improved performance in time management, task planning, and goal adherence, thereby reducing the risk of burnout. A bootstrap test indicated that Self-Control significantly mediates the relationship between physical exercise and Academic Burnout (mediating effect value = −0.013, 95% CI [−0.022, −0.004]). These findings offer a novel theoretical perspective and practical approach for intervening in adolescent Academic Burnout through Physical Exercise.

### 4.4 Mediating role of internet addiction and self-control

This study, based on the Protective-Risk Factors Model, systematically examines the dual mechanisms of Self-Control (a protective factor) and Internet Addiction (a risk factor) in the relationship between Physical Exercise and Academic Burnout. The results indicate that Internet Addiction and Self-Control not only have independent mediating effects between Physical Exercise and Academic Burnout (*β* = 0.354, *p* < 0.001, *E* = −0.021, 95% CI [−0.030, −0.013]; *β* = −0.504, *p* < 0.001, *E* = −0.013, 95% CI [−0.022, −0.004]) but also jointly form a chain mediation pathway of “Physical Exercise → Self-Control → Internet Addiction → Academic Burnout” (*E* = −0.0135, 95% CI [−0.0191, −0.0083]). This finding validates Hypothesis H4. According to the Strength Model of Self-Control, although Physical Exercise consumes a certain amount of psychological energy, this energy can be replenished and enhanced through recovery mechanisms, thereby strengthening an individual’s Self-Control capacity in a virtuous cycle (Tice et al., 2007). Empirical studies have shown that Physical Exercise significantly and positively predicts Self-Control levels (Du, 2024), which aligns with the findings of this study (Physical Exercise and Self-Control showed a positive correlation, *r* = 0.116, *p* < 0.01).

Within the framework of the Protective-Risk Factors Model, Self-Control, as a protective factor, can effectively buffer the negative impact of Internet Addiction, a risk factor. Specifically, individuals with high Self-Control levels are better able to regulate their smartphone use behaviors, reducing both the duration and frequency of internet use, thereby lowering the risk of Internet Addiction (Li et al., 2021). The study’s findings revealed that self-control had a notably stronger predictive impact on academic burnout (*β* = −0.695) compared to other variables. This influence remained substantial within the comprehensive model incorporating all variables (*β* = −0.504), underscoring self-control as the most potent protective factor against academic burnout. Consequently, interventions focusing on enhancing self-control are poised to deliver the most significant advantages. Additionally, a robust negative association emerged between Internet addiction and self-control (*r* = −0.494, *p* < 0.01, *β* = −0.849), alongside a noteworthy positive correlation between Internet addiction and academic burnout (*r* = 0.449, *p* < 0.01). These findings offer valuable insights into the intricate interplay among these three variables: physical activity could indirectly mitigate Internet addiction tendencies by enhancing self-control, subsequently diminishing the likelihood of academic burnout.

These findings endorse a hierarchical intervention strategy that prioritizes self-control cultivation as the core objective, employs physical exercise as a fundamental means, and identifies Internet addiction prevention as a critical focus area. Notably, while the direct effects of physical exercise may be limited, it can yield significant indirect benefits when systematically implemented as an intervention portal with robust operability.

## 5 Conclusion

This study utilized the Protective-Risk Factors Model and employed the Bootstrap method to systematically investigate the intrinsic relationships and mechanisms among Physical Exercise, Internet Addiction, Self-Control, and adolescent Academic Burnout. The primary conclusions are as follows:

### 5.1 Correlation analysis among variables

Significant correlations were observed between Physical Exercise, Internet Addiction, Self-Control, and adolescent Academic Burnout. Specifically, Physical Exercise was positively correlated with Self-Control and negatively correlated with Internet Addiction and Academic Burnout. Internet Addiction was positively correlated with Academic Burnout and negatively correlated with Self-Control. Self-Control was negatively correlated with Academic Burnout.

### 5.2 Independent mediating effects

Mediation analysis indicated that both Internet Addiction and Self-Control exerted significant independent mediating effects in the relationship between Physical Exercise and adolescent Academic Burnout. This finding further reveals a dual-pathway mechanism through which Physical Exercise influences Academic Burnout.

### 5.3 Chain mediating effect

Internet Addiction and Self-Control also functioned as chain mediators between Physical Exercise and adolescent Academic Burnout, forming the pathway: Physical Exercise → Self-Control → Internet Addiction → Academic Burnout. This result provides a new theoretical perspective for understanding the complex mechanism by which Physical Exercise affects Academic Burnout, while also supplying an empirical basis for intervening in adolescent Academic Burnout through Physical Exercise.

By introducing Academic Burnout into research on youth sports and school physical education, this study expands the research domain of Academic Burnout phenomena. From the perspective of school physical education, it explores a potential pathway for addressing issues related to adolescent Physical Exercise, academic performance, and psychological well-being. Furthermore, it offers empirical support for how schools can actively promote adolescents’ participation in Physical Exercise and help prevent Academic Burnout.

In light of China’s ongoing education reform efforts, this study proposes recommendations to enhance the physical and mental well-being of adolescents and mitigate academic burnout. Schools are advised to implement a structured physical exercise regimen by integrating regular sports activities into the weekly curriculum, such as incorporating 30-min morning exercises or extended recess periods daily, and instituting a “campus sports day” to ensure students engage in a minimum of 3 moderate-intensity workouts per week. This approach not only fosters health but also helps curb excessive screen time among students. Additionally, organizing time management workshops can facilitate the transfer of self-regulation skills honed through sports to academic pursuits. When designing the curriculum, adherence to the “learn, practice, compete” ethos is recommended, with the introduction of specialized modular courses alongside core subjects to diversify students’ options. Extracurricular offerings should encompass morning exercises, sports events, marathons, class competitions, and other activities, emphasizing engaging and challenging team projects to boost participation. Furthermore, incorporating Physical Exercise involvement and sportsmanship into students’ comprehensive quality assessment, establishing a collaborative framework between physical education instructors and class teachers, monitoring students’ physical and mental well-being, and offering tailored sports regimens for those displaying signs of Academic Burnout are recommended. These strategies contribute to fostering a wholesome campus sports culture, boosting students’ psychological fortitude and self-regulation skills, ultimately mitigating academic burnout and fostering holistic development.

This study has several limitations. First, while the constructed mediation model aligns with the causal theoretical framework, the cross-sectional design inherently limits the establishment of precise causal relationships. It is essential to recognize that inverse causal relationships may exist between variables; for instance, individuals with lower self-control may engage less in physical activity. Future research could employ longitudinal tracing designs, such as cross-lag panel analysis, or experimental intervention methods to more accurately verify the causal pathways and mechanisms linking physical exercise, self-control, Internet addiction, and academic burnout.

Second, this study focused solely on the chain mediation between self-control and Internet addiction. Subsequent studies should incorporate additional relevant variables, such as personality traits and lifestyle factors closely associated with academic burnout, to elucidate the internal mechanisms through which physical exercise influences academic burnout from multiple perspectives.

Third, the participant sample in this study was restricted to typical middle school students, excluding those from vocational or sports schools. Similar relationships may be present among different student populations. Therefore, future studies should broaden the sample size to enhance the representativeness and generalizability of the findings.

Hence, future research can be further expanded in several directions. First, it is advisable to adopt a longitudinal research design, such as cross-lag panel analysis, to investigate the dynamic interaction between physical exercise and academic burnout through long-term follow-up. Second, randomized controlled experiments should be designed to test the causal relationships between variables by systematically increasing physical exercise interventions among students. Additionally, it is essential to validate the theoretical model proposed in this study across groups with diverse cultural backgrounds and age stages, such as primary school and university students, to assess its universality. Concurrently, researchers should utilize existing data or expand the sample size to explore the moderating roles of variables such as gender and school type within the model. For instance, analyzing whether significant differences exist in mediation paths between male and female students or across different grade groups would be beneficial. Finally, future studies could incorporate additional relevant variables, such as psychological resilience, social support, and stress response, to elucidate the complex mechanisms through which physical exercise influences academic burnout from multiple dimensions. By pursuing these specific research avenues, scholars can contribute to a more comprehensive theoretical framework and provide targeted practical guidance for various student populations.

## Data Availability

The raw data supporting the conclusions of this article will be made available by the authors, without undue reservation.
