# Supplementary material for: The relationship between physical exercise and academic burnout in adolescents: the chain-mediated role of internet addiction and self-control
Source: Front Psychol. 2025 Nov 27;16:1710564. doi: 10.3389/fpsyg.2025.1710564 (PMC12695792; doi:10.3389/fpsyg.2025.1710564)
Supplement: Supplementary file 1 [file Data_Sheet_1.pdf]

Supplementary Material

1 Supplementary Tables

Table S1.
Spearman rank correlation coefficient matrix between variables

Variable	*M*±*SD*	1	2	3	4
Physical Exercise (1)	3.037±0.779	1	-	-	-
Internet Addiction (2)	1.894±0.620	-0.104**	1	-	-
Self-Control (3)	3.317±0.353	0.127**	-0.499**	1	-
Academic Burnout (4)	2.296±0.497	-0.121**	0.450**	-0.519**	1

Note: $N = 1948$;*$p < 0.05$， **$p < 0.01$。

Table S2.
Parameter Estimation of Generalized Linear Model Based on Robust Standard Error

Predictor variables	B	Robust standard error	Waldkafang	*p*
(intercept)	3.641	0.145	634.161	< 0.001
Physical Exercise	-0.028	0.012	5.089	0.024
Internet Addiction	0.217	0.020	118.417	< 0.001
Self-Control	-0.504	0.034	216.141	< 0.001

Note: Dependent variable is Academic Burnout (Y): model likelihood ratio chi-square = 705.393(3), degree of freedom = 3, $p < 0.001$.

This section presents the results of two sensitivity analyses to test the robustness of the main study findings. Table S1 presents Spearman rank correlation matrices for all key variables, with correlation patterns that are highly consistent with Pearson correlation matrices reported in the main analysis, indicating that the associations between variables are not strictly constrained by linear and normal distribution assumptions. Table S2 shows the results of the generalized linear model re-estimated using robust standard error (HCO). The direction, size and statistical significance of the regression coefficients of each key predictor are completely consistent with the ordinary least squares regression results of the main analysis, demonstrating that the study findings are not affected by heteroskedasticity. Combining the two analyses, the conclusions of the main study are robust.
